# Comparison of stethoscope bell and diaphragm, and of stethoscope tube length, for clinical blood pressure measurement

**DOI:** 10.1097/MBP.0000000000000175

**Published:** 2016-01-06

**Authors:** Chengyu Liu, Clive Griffiths, Alan Murray, Dingchang Zheng

**Affiliations:** a Institute of Cellular Medicine, Newcastle University, Newcastle upon Tyne; b Health & Well Being Academy, Faculty of Medical Science, Anglia Ruskin University, Chelmsford, UK; c School of Control Science and Engineering, Shandong University, Jinan, China

**Keywords:** bell and diaphragm, blood pressure, Korotkoff sound, stethoscope characteristic, stethoscope tube length

## Abstract

**Objective:**

This study investigated the effect of stethoscope side and tube length on auscultatory blood pressure (BP) measurement.

**Methods:**

Thirty-two healthy participants were studied. For each participant, four measurements with different combinations of stethoscope characteristics (bell or diaphragm side, standard or short tube length) were each recorded at two repeat sessions, and eight Korotkoff sound recordings were played twice on separate days to one experienced listener to determine the systolic and diastolic BPs (SBP and DBP). Analysis of variance was carried out to study the measurement repeatability between the two repeat sessions and between the two BP determinations on separate days, as well as the effects of stethoscope side and tube length.

**Results:**

There was no significant paired difference between the repeat sessions and between the repeat determinations for both SBP and DBP (all *P*-values>0.10, except the repeat session for SBP using short tube and diaphragm). The key result was that there was a small but significantly higher DBP on using the bell in comparison with the diaphragm (0.66 mmHg, *P*=0.007), and a significantly higher SBP on using the short tube in comparison with the standard length (0.77 mmHg, *P*=0.008).

**Conclusion:**

This study shows that stethoscope characteristics have only a small, although statistically significant, influence on clinical BP measurement. Although this helps understand the measurement technique and resolves questions in the published literature, the influence is not clinically significant.

## Introduction

Manual auscultatory blood pressure (BP) measurement is widely recommended as the gold standard for noninvasive clinical BP measurement. It is the most accurate technique for routine BP measurement 1–3; it requires a cuff, a stethoscope, and a cuff pressure display. A trained observer uses a stethoscope to listen for the Korotkoff sounds associated with blood flow through the brachial artery as a BP cuff encircling the upper arm is deflated 4. The appearance and disappearance of Korotkoff sounds is associated with systolic and diastolic BPs (SBP and DBP), respectively, and the BPs at these times are read from a cuff pressure display.

A stethoscope usually consists of a bell, a diaphragm, a tube, and earpieces. Either the stethoscope bell or the stethoscope diaphragm is used for capturing the appearance and disappearance of Korotkoff sounds during cuff deflation. The common viewpoint is that the stethoscope bell would perform better in recording Korotkoff sounds with a low frequency range, whereas the stethoscope diaphragm would perform better with a high frequency range 5,6. The study published by Abella *et al*. 6 showed that the stethoscope bell provided a louder output than the diaphragm at the low frequency range, and thus they suggested that the stethoscope bell outperforms the diaphragm in recording heart sounds. The stethoscope diaphragm was recommended when high-frequency components of heart or Korotkoff sounds were required 5. However, ambiguous recommendations for selecting the stethoscope bell or diaphragm side for BP measurement have been provided in different textbooks. Among the recommendations published over the past 20 years, two recommended the stethoscope bell 7,8, three the stethoscope diaphragm 9–11, and three the bell and/or diaphragm 12–14. International BP measurement guidelines also gave diverse recommendations. Both the 1997 Sixth Report of the Joint National Committee on Prevention, Detection, Evaluation, and Treatment of High Blood Pressure 15 and the 1999 WHO – International Society of Hypertension guidelines 16 recommended the stethoscope bell, because it is believed that the Korotkoff sounds mainly contain low-frequency components. The 2003 Seventh Report of the Joint National Committee 17 did not state specifically which stethoscope side should be used. However, the 2003 European Society of Hypertension guidelines 18 recommended the use of the diaphragm side because it is easier to hold and covers a greater skin contact area.

The importance of accurate BP measurement in clinical practice is without doubt, and small inaccuracies in BP measurement can have considerable consequences 19. It has been reported by population studies that overestimating or underestimating BP by even 5 mmHg can seriously compromise diagnosis, resulting in millions of people being wrongly diagnosed as hypertensive, with attendant exposure to adverse drug effects, or being denied treatment, leading to associated cardiovascular conditions, including fatal stroke and fatal myocardial infarction 20,21. Therefore, any potential small BP difference caused by stethoscope characteristics is clinically important and worth further investigation.

In real clinical practice, both bell and diaphragm sides of the stethoscope are commonly used. Obtaining the sounds from different stethoscope sides may generate different ways for enabling Korotkoff sounds to be heard, resulting in different interpretations by observers and hence different BP readings. In addition, stethoscope tube lengths vary from 55 to 80 cm, but they usually have a length of 70 cm, which is simply referred to as ‘standard tube’ in this study. However, the influence of stethoscope length on BP readings has not been quantitatively investigated.

Thus, the aims of the current study were to quantify the BP difference between the measurements undertaken using the stethoscope bell and diaphragm sides, and between those undertaken using different tube lengths.

## Methods

### Participants

The required sample size was estimated from a power calculation allowing a 5 mmHg mean BP difference to be detected with a typical 8 mmHg SD of BP measurement; 21 participants were required to achieve a confidence level of 95% and a statistical power of 80%. Thirty-two healthy participants (19 male and 13 female) were recruited from May to July 2014, with ages ranging from 24 to 68 years. They were mainly from among the staff, students, and visitors of Freeman Hospital and Newcastle University. Exclusion criteria for this study were age under 18 years or over 70 years, known cardiovascular disease including atrial fibrillation or other irregular heart rhythms, and pregnancy.

This study received ethical approval from the Newcastle & North Tyneside Research Ethics Committee. The investigation conformed to the principles of the Declaration of Helsinki. All participants gave their written informed consent to participate in the study. Table 1 briefly summarizes the demographic information of the participants, including sex, age, height, weight, and arm circumference.

**Table 1:**
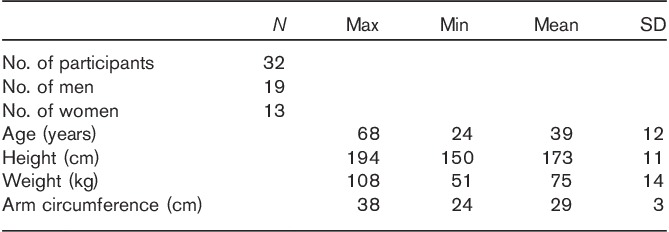
General information for the participants studied

### Korotkoff sound recording

All BP measurements were performed in a quiet and temperature-controlled clinical measurement room by a trained operator at the Freeman Hospital, Newcastle upon Tyne, UK. Before the formal recording, each participant was asked to rest on a chair for 5 min. BP measurements were performed with the participant in a sitting position, with his/her feet placed on the floor and the arm supported at the level of the heart. For signal recording, we located the stethoscope head at the position with the maximum pulse beat obtained with moderate applied pressure. The analog sound signals were then recorded to a computer from an audio amplifier with a constant gain for all recordings. This gain had been set in a preliminary study. No recorded signal saturated the recording range. The participants were also asked to breathe gently during the measurement. The whole procedure followed the guidelines recommended by the British Hypertension Society and American Heart Association 2,22.

**Fig. 1:**
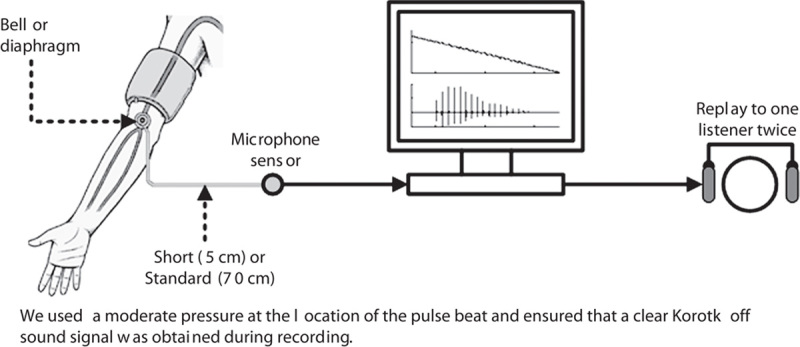
Diagram of the blood pressure measurement system for digitally recording Korotkoff sounds. Four different combinations of stethoscope characteristics are illustrated.

Figure 1 shows a diagram of the BP measurement system with four different combinations of stethoscope characteristics: bell plus standard tube (70 cm), bell plus short tube (5 cm), diaphragm plus standard tube, and diaphragm plus short tube. The tubes used were rubber tubes with an inner diameter of 2.4 mm and a thickness of about 0.25 mm. For each participant, there were two repeat sessions with four measurements for each, giving a total of eight recordings. There was a time interval of at least 1 min between the four measurements within a session and at least 4 min between the two sessions, allowing recovery of cardiovascular hemodynamics. The order of the four measurements within the sessions for each participant was randomized. During cuff deflation, the cuff pressure and Korotkoff sounds were digitally recorded at a sample rate of 2000 Hz. The cuff pressure was linearly deflated at a standard rate of 2–3 mmHg/s. The deflation rate was automatically controlled.

### Blood pressure determination

For each participant, eight recordings of Korotkoff sounds (from two repeat sessions and four stethoscope combinations) were converted into .wav files using Matlab 2011a (MathWork Inc., Natick, Massachusetts, USA). Because of the potential BP measurement bias from repeat BP determinations 23–25, all Korotkoff sounds recorded in this study were replayed twice (on two different days) to one trained listener to test the repeatability of BP determination. The observer was well trained and certified using the British Hypertension Society’s Blood Pressure Measurement educational tool and supporting material. The order of replaying all the 256 Korotkoff sound recordings (from eight Korotkoff recordings for each participant×32 participants) was randomized, and the listener was unaware of any participant or combination information. All BP measurements and microphone signal playbacks were performed in a quiet, temperature-controlled clinical measurement room. We recorded the background noise level in this room, and the noise level was usually below 30 dB when performing the signal playback. The same microphone amplifier and computer settings were used throughout the study to ensure that the playback was exactly the same for every participant on all days. The listener identified the pressure associated with the appearance and disappearance of the sounds for the determination of SBP and DBP by reading the cuff pressure display, similar to a mercury column. BP values were determined to an accuracy of 2 mmHg.

### Data and statistical analysis

In total, 16 values were obtained from each participant (from two stethoscope sides, two tube lengths, two repeat measure recordings, and two BP determinations on separate days) for both SBP and DBP. The overall mean and SD of the BPs were calculated across all participants, as well as separately for the two stethoscope sides, two tube lengths, and their combinations.

The SPSS Statistics 19 software package (SPSS Inc., Chicago, Illinois, USA) was used to carry out analysis of variance to study the repeatability of measurements between the two repeat sessions and between the two BP determinations on separate days, as well as the effects of stethoscope side and tube length. The mean BP differences between the above factors were also analyzed. All differences were paired values, and a *P*-value less than 0.05 was considered as a statistically significant difference.

## Results

### Blood pressures

Among all 32 participants, the mean±SD BP calculated from all data was 109.9±12.3 mmHg for SBP and 71.2±9.3 mmHg for DBP. The results for the separate stethoscope characteristics are shown in Table 2.

**Table 2:**
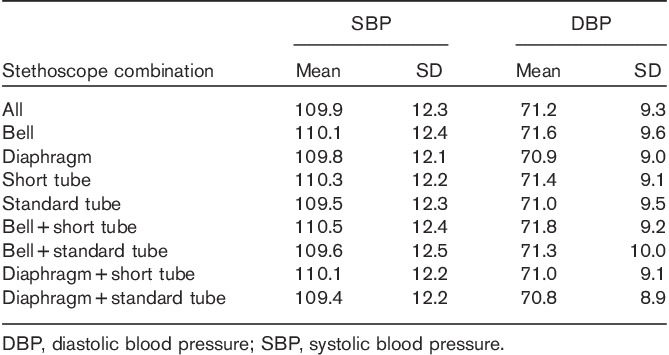
Blood pressure results (mmHg) from all data, as well as from the separate stethoscope side and tube length

### Repeat measurements

There was only one significant paired difference between the repeat measurement sessions for SBP and DBP (all *P*-values>0.10, except the SBP difference measured using the short tube and diaphragm 1.7±3.5 mmHg, *P*=0.01). The overall SBP and DBP changes between the two measurement sessions were 0.91±4.72 and −0.07±3.74 mmHg.

### Repeat listening

**Fig. 2:**
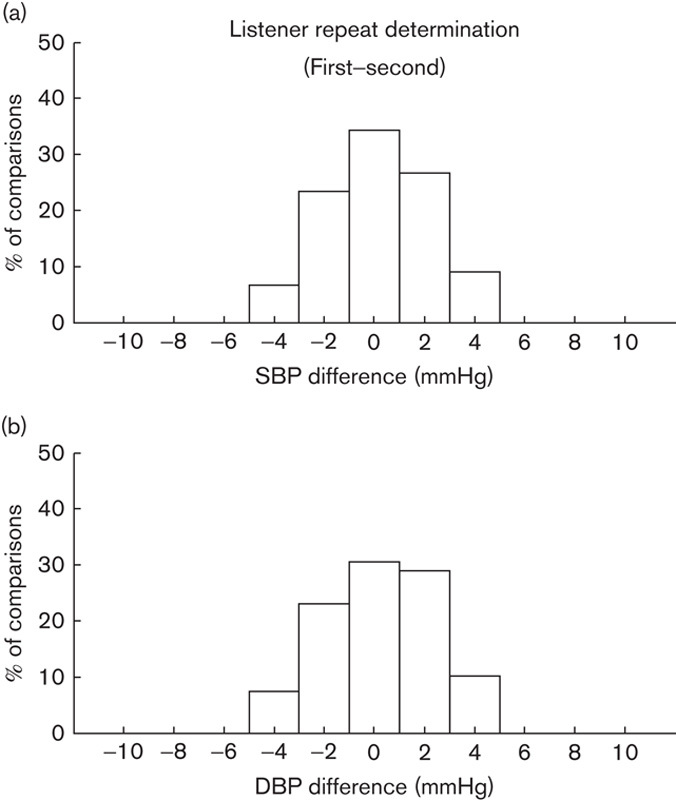
Histogram of within-subject (a) SBP and (b) DBP differences between repeat determinations on separate days (first minus second determination). A total of 256 comparisons (from 32 participants, two stethoscope sides, two tube lengths, and two repeat measurement sessions) were made for both SBP and DBP. DBP, diastolic blood pressure; SBP, systolic blood pressure.

Figure 2 shows histogram of within-subject SBP and DBP differences between the repeat determinations on separate days. There was no significant paired difference between the repeat listening results for both SBP (0.16±2.12 mmHg, *P*=0.24) and DBP (0.23±2.20 mmHg, *P*=0.11). As the order of the repeat listening was randomized and the repeat listening was on another day, our results confirmed the accuracy of BP determination for the data in this study.

### Effect of bell or diaphragm

**Fig. 3:**
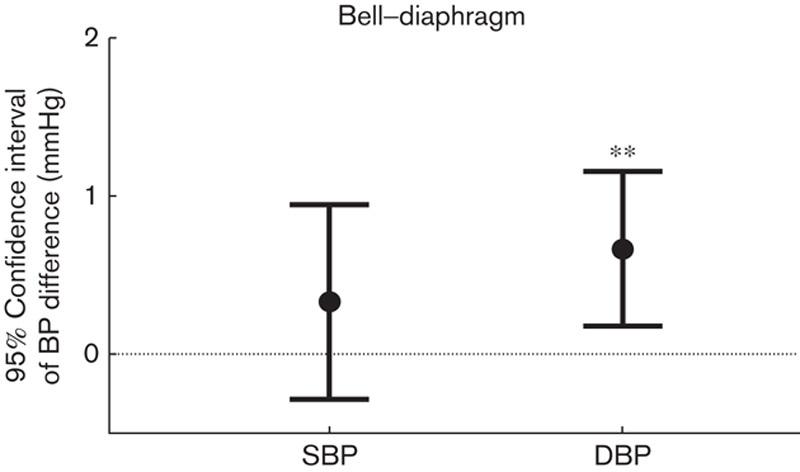
Error chart with 95% confidence interval of the within-subject SBP and DBP differences between bell and diaphragm sides. **Significant difference, *P*<0.01. DBP, diastolic blood pressure; SBP, systolic blood pressure.

All results showed a tendency toward higher BP values with the bell in comparison with the diaphragm, and this was statistically significant for DBP (mean difference 0.66 mmHg, 95% confidence interval 0.18–1.15 mmHg, *P*=0.007; Fig. 3).

### Effect of tube length

**Fig. 4:**
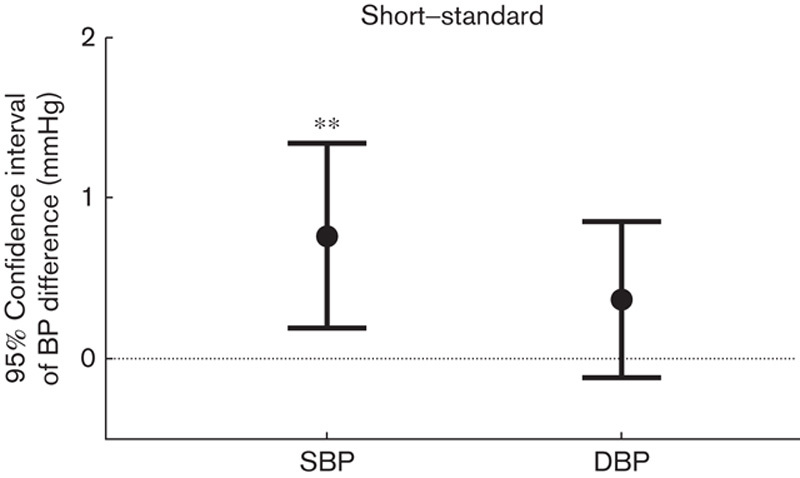
Error chart with 95% confidence interval of the within-subject SBP and DBP differences between short and standard tube lengths. **Significant difference, *P*<0.01. DBP, diastolic blood pressure; SBP, systolic blood pressure.

All results showed a tendency toward higher BP values with the short tube in comparison with the standard length tube, and this was statistically significant for SBP (mean difference 0.77 mmHg, 95% confidence interval 0.20–1.33 mmHg, *P*=0.008; Fig. 4).

## Discussion

Our study has quantitatively shown that BPs measured with stethoscopes of different characteristics had small differences. A significantly higher DBP of 0.66 mmHg was observed on using the bell in comparison with the diaphragm, and a significantly higher SBP of 0.77 mmHg was observed on using the short tube in comparison with the standard tube. To the best of our knowledge, this is the first clinical study to compare the BP difference from the different combinations of stethoscope sides and tube lengths. These BP differences provide evidence that using any stethoscope combination results in a very small, although statistically significant, influence on clinical BP measurement.

Clinical studies aiming to compare BP differences between stethoscope bell and diaphragm measurements have reported different conclusions. The study by Mauro 26 demonstrated a higher SBP and a lower DBP with the stethoscope bell in comparison with the diaphragm. Prineas and Jacobs 27 reported from 48 supine adults that using the stethoscope bell over the brachial artery gave significantly higher values for both SBP and DBP than using the diaphragm side on the cubital fossa. However, the study by Kantola *et al*. 28 of 250 hospital inpatients showed that both stethoscope sides gave similar results with the acoustic BP measurement, but there were significant differences when using either low-frequency or high-frequency amplification for Korotkoff sounds. The study by Cushman *et al*. 29 of 48 men with histories of primary hypertension also reported that there was no significant SBP and DBP difference between the bell and the diaphragm. Our study demonstrated a tendency toward higher BP values with the bell in comparison with the diaphragm, with a significantly higher DBP. This interesting conclusion requires a better understanding of how the stethoscope side influences clinical BP measurement, such as with hypertensive or other cardiovascular patients, to obtain a wide range of BP readings and a wide range of heart rate readings. Because there was no clinically significant difference between the bell and diaphragm, the recommendation by O’Brien *et al.* 18 to use the diaphragm is reasonable because of the easier placement of the diaphragm side on the antecubital fossa.

With regard to the effect of tubing on BP measurement and Korotkoff sound features, Rappaport and Sprague 30 investigated the physical properties and its effect on stethoscope efficiency. Ertel *et al*. 31 reported that using a double or single stethoscope tube influenced the transmission patterns of the Korotkoff sounds, resulting in different BP determinations. However, to the best of our knowledge, this is the first scientific quantitative evidence on the effect of stethoscope tube length on BP measurement. Although this mean SBP difference of 0.77 mmHg is considered to be statistically significant, it is not clinically important. It does, however, resolve some uncertainty in the published literature.

In addition, Hampton and Chaloner 32 reported that deep inspiration could influence the stethoscope performance, and accurate positioning of the stethoscope head on the chest wall was essential for clear sound collection, especially for lower frequency components. In the present study, we located the stethoscope head at the position with the maximum pulse beat and obtained sound signals with moderate applied pressure. During measurement, we also asked the participants to breathe gently to reduce the influence of respiration.

One possible explanation for the lower DBP from the diaphragm is that the diaphragm side could respond better to the high-frequency component of Korotkoff sounds. This has been reported by several published studies 5,9–11. One possible explanation for the higher SBP from the short tube is that, during cuff pressure deflation, arterial flow is heard more easily using the short tube length, resulting in the systolic Korotkoff sounds from the stethoscope with the short length tube appearing earlier than those with the standard length tube.

Some potential limitations should be addressed. First, the recorded Korotkoff sounds were played back to a single observer. However, the auditory acuity of the observer was checked. In addition, the recorded Korotkoff sounds were replayed twice (on two different days) to the trained investigator to further verify BP determination, and the results showed that repeatability was very good. Further, a similar study previously conducted by us using two observers showed no significant difference between them 3. Therefore, we are confident that the observation can be extended to all observers, provided they are properly trained for clinical BP measurement using the traditional auscultatory method. Moreover, the selection of cuff followed the BP measurement guidelines, with the participants’ arm circumference falling in the central 75% of the cuff’s range. We used the standard BP cuffs from AC Cossor & Son Ltd (Harlow, Essex, UK). The adult cuff (maximum arm circumference 34.3 cm) and alternative adult cuff (maximum arm circumference 42.0 cm) were used according to the arm circumference of the participant (Table 1). The cuffs were latex-free. Finally, we note that automated devices are leading to a decline in manual measurement, but the manual technique is still the gold standard for accurate clinical measurement and for the evaluation of automated devices in the various international standards.

In summary, the effects of stethoscope side and tube length on BP measurements have been quantified, providing scientific evidence that stethoscope characteristics have only a small, although statistically significant, influence on clinical BP measurement. However, this influence is not clinically significant.
